# Abnormalities in Copper Status Associated with an Elevated Risk of Parkinson’s Phenotype Development

**DOI:** 10.3390/antiox12091654

**Published:** 2023-08-22

**Authors:** Marina N. Karpenko, Zamira M. Muruzheva, Ekaterina Yu. Ilyechova, Polina S. Babich, Ludmila V. Puchkova

**Affiliations:** 1 I.P. Pavlov Department of Physiology, Research Institute of Experimental Medicine, 197376 St. Petersburg, Russia; mnkarpenko@mail.ru (M.N.K.); zamira.muruzheva@mail.ru (Z.M.M.); 2 Institute of Biomedical Systems and Biotechnology, Peter the Great St. Petersburg Polytechnic University, 195251 St. Petersburg, Russia; puchkovalv@yandex.ru; 3 State Budgetary Institution of Health Care “Leningrad Regional Clinical Hospital”, 194291 St. Petersburg, Russia; 4 Research Center of Advanced Functional Materials and Laser Communication Systems, ADTS Institute, ITMO University, 197101 St. Petersburg, Russia; 5 Department of Molecular Genetics, Research Institute of Experimental Medicine, 197376 St. Petersburg, Russia; 6 Department of Zoology and Genetics, Faculty of Biology, Herzen State Pedagogical University of Russia, 191186 St. Petersburg, Russia; babich.polina@gmail.com

**Keywords:** Parkinson’s disease, serum copper level, serum ceruloplasmin, cerebrospinal fluid, meta-analysis

## Abstract

In the last 15 years, among the many reasons given for the development of idiopathic forms of Parkinson’s disease (PD), copper imbalance has been identified as a factor, and PD is often referred to as a copper-mediated disorder. More than 640 papers have been devoted to the relationship between PD and copper status in the blood, which include the following markers: total copper concentration, enzymatic ceruloplasmin (Cp) concentration, Cp protein level, and non-ceruloplasmin copper level. Most studies measure only one of these markers. Therefore, the existence of a correlation between copper status and the development of PD is still debated. Based on data from the published literature, meta-analysis, and our own research, it is clear that there is a connection between the development of PD symptoms and the number of copper atoms, which are weakly associated with the ceruloplasmin molecule. In this work, the link between the risk of developing PD and various inborn errors related to copper metabolism, leading to decreased levels of oxidase ceruloplasmin in the circulation and cerebrospinal fluid, is discussed.

## 1. Introduction

Parkinson’s disease (PD) is a chronic neurodegenerative illness that affects older people. Currently, PD affects approximately 0.3% of the global population and affects up to 5% of people aged over 80 [1]. PD is ranked second in occurrence among neurodegenerative diseases (following Alzheimer’s disease) and contributes to the majority (almost 80%) of cases that are collectively referred to as parkinsonism or parkinsonian syndrome. Apart from diagnosed PD, this group also includes other motion-related illnesses, including progressive supranuclear palsy, multiple system atrophy, dementia with Lewy bodies, etc. [2,3]. The major clinical symptoms of this group of diseases are motor symptoms (bradykinesia, muscular rigidity, resting tremors, and postural instability), which are usually preceded by non-motor symptoms (behavioral/neuropsychiatric changes, autonomic nervous system failure, hyposmia, and sleep disturbances) that reflect the ongoing changes in the central and peripheral nervous systems [4,5]. Both motor and non-motor symptoms invariably progress over time, with the subsequent accession of cognitive and mental disorders.

The death of dopamine-producing neurons in the pars compacta region of the substantia nigra (SN) is invariably observed in PD patients, irrespective of the external symptoms. This process is manifested by the discoloration of brain tissue, due to the loss of neuromelanin [6]. Another specific trait of PD is the oligomerization of α-synuclein in β-sheet-rich amyloid fibrils, which form “Lewy bodies”—dense, spherical intracellular protein aggregates with a characteristic peripheral halo [2,7].

Almost 90% of cases of PD are considered idiopathic, caused by various adverse behavioral and environmental factors (including, but not limited to, lifestyle, obesity, exposure to pesticides, medications, compounds of transition metal ions that can catalyze Fenton-type reactions, viral and bacterial infections, and oxidative stress) [8,9,10,11,12,13,14,15,16]. Age, gender, race, ethnicity, low levels of physical activity, and various metabolic disorders can worsen the effects of environmental factors [13,14,15,16,17,18,19,20,21].

About 3–10% of PD cases are family cases exhibiting Mendelian inheritance. Both dominant and recessive PD-related alleles are known, but all the studied genes display incomplete penetrance. Extensive data on the correlation between mutations in these genes and the PD phenotype have been widely analyzed in modern reviews published in high-impact journals [18,22,23,24,25,26,27,28]. The genes under investigation, in which mutations are definitely linked to the development of family PD cases, are not very numerous; these genes are listed in Table 1.

Table 1 draws attention to the fact that the products of the listed genes are involved in the control of various types cellular fundamental processes: nonselective vesicular transport, the endo/autolysosomal system, and proteasomal degradation, along with quality control and the disposal of mitochondria. Not surprisingly, mutations in these genes globally disrupt the transmission and elimination of denatured proteins and damaged organelles, which have lost their physiological functions; this behavior is characteristic of both family and sporadic PD cases. There are reports on the less pronounced association between the development of the PD phenotype and mutations in other genes. These include the genes responsible for control of the assembly of the respiratory electron transport chain complexes I, III, and IV, the neutralization of xenobiotics, etc. [29,30,31,32].

Conversely, genome-wide association studies of sporadic PD cases have revealed more than 200 genes in which mutations can potentially lead to the development of the PD phenotype [18]. As a result, an argument is gaining ground that many idiopathic PD cases are actually caused by mutations in genes that have not yet been identified as PD-associated mutations/genes [29].

The importance of environmental factors in the pathogenesis of PD is supported by large-scale population studies that were carried out on 19,842 twins. These studies have shown that the probandwise concordance rate comprised 0.20 and 0.13 in homozygous and dizygous pairs, respectively. The high concordance rate in dizygous twins favors the concept that environmental factors in early childhood are more important for PD development than the presence of genetic factors [33,34]. Environmental factors, the significance of which in PD development has been supported by experimental and population studies, include exposure to various metal ions: mercury, lead, manganese, copper, iron, aluminum, bismuth, thallium, and zinc [35]. It is easy to see that this list contains both abiogenic elements and essential micronutrients. Abiogenic ions, which can mimic the essential metal ions and intervene in normal biochemical processes, are especially toxic [36,37,38,39,40]. For example, mercury, lead, bismuth, and thallium are well known as xenobiotics. They disrupt the body’s intermolecular interactions and catalytic activities, displace essential metal ions, facilitate protein aggregation, and ultimately provoke oxidative stress and reactive oxygen species (ROS) formation. Oxidative stress impairs the normal functioning of the proteasome system, leading to the accumulation of improperly folded proteins in the cytosol [41]. High levels of protein aggregation, augmented by direct and indirect DNA damage from toxic metals, are fatal to cellular and mitochondrial metabolism and eventually lead to apoptosis. In particular, the death of dopaminergic neurons in the brain results in various neurodegenerative disorders characteristic of PD.

**Table 1:** Genes with mutations that are strongly associated with the risk of PD-like phenotype development.

No	Gene	Protein Product and Its Function	Functions	Phenotype at PD	Ref.
1.	*SNCA*	α-synuclein	Neuron synaptic vesicle trafficking and neurotransmitter release	Oligomerization of α-synuclein in β-sheet-rich amyloid fibrils; the formation of Lewy bodies	[42,43]
2.	*PINK1*	PTEN-induced putative kinase 1 (mitochondrial serine/threonine-protein kinase 1)	Marks mitochondria for their elimination through mitophagy	Impaired quality control of mitochondria	[31]
3.	*PARK2*	E3 ubiquitin ligase	Takes part in mitophagy and ubiquitin-dependent proteasomal degradation	Impaired quality control of mitochondria	[26]
4.	*PARK7*	deglycase DJ-1	Controls the Ca^2+^ influx to the mitochondria and supports α-synuclein structure due to chaperone activity; possesses antioxidant properties	High cytosolic levels of catecholamines and Ca^2+^ ions	[44]
5.	*LRRK2*	Leucine-rich repeat kinase 2	Takes part in chaperone-mediated autophagy and mitophagy	Impaired quality control of cytosolic proteins and mitochondria	[31,45,46]
6.	*VPS35*	vacuolar protein sorting-35	Plays a role in endosomal—trans-Golgi transport and membrane recycling	Impaired endosomal recycling	[47,48]
7.	*GBA1*	lysosome glycosylceramidase beta	Cleaves the β-glucosidic linkage of glycosylceramide	Lysosomal dysfunction, reduced contacts between mitochondria and endoplasmic reticulum, and induced stress of endoplasmic reticulum	[49,50]
8.	*PLA2G6* *(PARK14)*	enzyme (iPLA2)	Hydrolyzes phospholipids, generating free fatty acids and lysophospholipids	Impaired structure and function of the lipid bilayer in the membranes of astrocytes	[51]

Iron, copper, and manganese are essential trace elements; they are ubiquitous micronutrients that must be absorbed by the organism along with food or water [52]. In all phyla of living organisms, the deficiency of these elements is incompatible with normal vital activity [53]. Ions of these elements can catalyze Fenton-type reactions and ROS formation in a similar way to many abiogenic transition metals. Therefore, all organisms possess evolutionarily conserved systems of transport for proteins that enable the safe trafficking of metal ions. The main principle of these systems is the constant retention of the ions in the coordination spheres of the proteins. The systems include membrane and soluble transporters that control the amount of trace elements absorbed from food; they also provide safe ion trafficking to the formation sites of metal-containing enzymes or metal-dependent regulatory proteins. The cellular and organismal balance of each trace element is maintained by a specific homeostatic system [54,55,56,57,58].

In a natural environment, an excess of micronutrients is a relatively rare occurrence; however, there are geochemical niches and areas in which life quality is limited because of trace element deficiency [59]. However, in one study, a correlation between the consumption of copper, manganese, iron, and magnesium with food and the risk of PD development was not observed [60]. At the same time, toxic excesses or deficiencies of such microelements inevitably result from genetic defects in the transporter proteins, which maintain ion homeostasis [61,62,63,64]. This is especially true in the case of copper homeostasis systems and PD development. It has also been pointed out that iron accumulation in the SN is one of the major pathognomonic traits of PD [65,66]. Iron accumulation is usually associated with a deficiency of the copper-containing ferroxidases that provide bidirectional copper transport through biological membranes [67,68,69]. The aim of this article is to discuss the association of abnormalities in copper metabolism with the risk of PD development.

## 2. Copper’s Physiological Function and Its Safe Turnover in the Body

### 2.1. Biological Role of Copper

Copper belongs to the group of essential trace elements and typically ranks third in terms of its specific content in living organisms (after iron and zinc). In mammals, copper is present in the active sites of enzymes, which take part in respiration, ROS detoxification, connective tissue formation, transmembrane iron transport, erythropoiesis, neurotransmitters synthesis and degradation, post-translational maturation of neuropeptides, hormones, sulfated sugars, etc. [70,71,72]. In addition, copper demonstrates the properties of a secondary messenger. Thus, it takes part in signaling [73,74,75,76], regulates gene activity through its binding to copper-dependent transcription factors [77,78,79,80,81,82,83], and modulates enzyme activity as an allosteric effector [84]. It also serves as a cofactor of some receptors [85,86], controls mitophagy [74,87], and causes cuproptosis, copper-dependent mitochondria-mediated programmed cell death [88]. In the nervous system, copper ions function as signaling agents [89]. They are released from the synaptic terminals of the neurons, affecting the postsynaptic receptors, and regulate neuronal excitability [75,90]. At the same time, as discussed above, copper can be a highly toxic agent [72].

### 2.2. Mechanisms for the Safe Use of Copper Ions as Enzymatic Cofactors and Their Delivery to Cell Compartments

**Figure 1:**
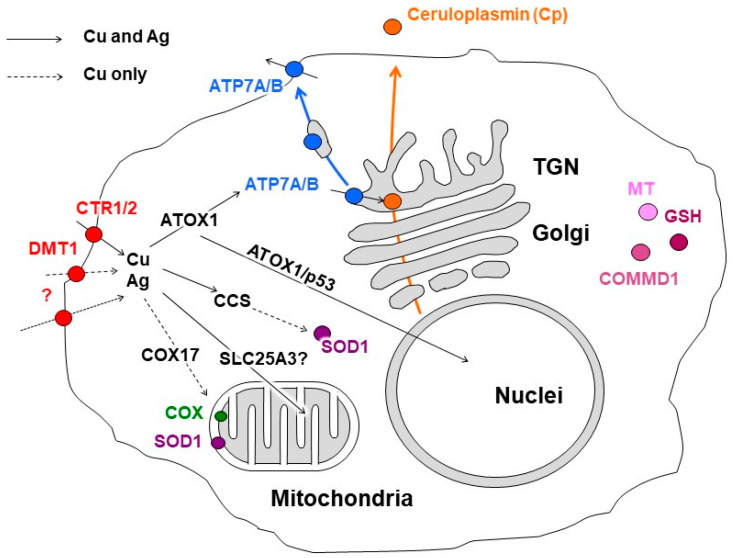
Scheme of copper distribution in a mammalian cell. Copper is taken up via copper transporter 1 (CTR1), divalent metal transporter 1 (DMT1), or a putative transporter (all depicted as red circles). After being imported into the cell, copper is transferred to the chaperones, antioxidant protein 1 (ATOX1), copper chaperone (CCS), and cytochrome-*c*-oxidase copper chaperone (COX17), which ferry the copper (indicated by black arrows) to copper-transporting ATPase (ATP7A/B, shown in blue) in the Golgi complex, to Cu,Zn-superoxide dismutase (SOD1, shown in magenta) in the cytosol, and to cytochrome-*c*-oxidase (COX, shown in green) in the mitochondria. The mitochondrial phosphate carrier protein (SLC25A3) transfers the copper into the matrix. In the Golgi complex, the ATP7A/B loads the copper onto newly synthesized cuproenzymes, such as ceruloplasmin (Cp, shown in orange circle), which transport it along the biosynthetic pathway (indicated by orange arrow). A significant increase in intracellular Cu induces the export of ATP7A/B (indicated by blue arrow) toward the post-Golgi compartments (TGN) and plasma membrane, where it drives the excretion of excessive copper from the cell. The copper metabolism MURR1 domain protein 1 (COMMD1) is involved in copper transport and protein trafficking/degradation. Excessive copper could bind to cysteine-rich proteins, known as metallothioneins (MTs, shown in pink). The scheme is modified from our article [38].

Enzymes that utilize copper as a cofactor bind to it with high affinity. In such binding sites, the polypeptide chain provides 4–5 donor groups to the ion, preventing the loss of the metal during the redox cycling of copper [91]. Conversely, metal ions transporting proteins act to transfer metal, either by having low-affinity binding sites with a moderate coordination number of ~4, or by having lower coordinate binding sites of 2–3 ligands that bind with high affinity. Both strategies retain the metal but allow its transfer under the appropriate conditions [91,92]. Cu-chaperones use the second strategy. They bind copper ions with low coordination numbers and pass the copper ions to each other via direct protein–protein interactions, forming a group of dedicated proteins related to copper metabolic systems (CMS) (Figure 1). As a result, there are no “free” copper ions in the cell [93]. However, any defects in the CMS protein structure or in the cuproenzymes’ active sites can result in the leakage of copper ions and the subsequent disturbance of cell metabolism [94]. The operation of intracellular CMS is neatly integrated with extracellular copper-trafficking pathways. In mammals, the interaction of intracellular CMS and body copper-transport systems enables safe copper transition in the organism.

### 2.3. Machinery for the Conversion of Dietary Copper to Catalytic and Regulatory Copper

**Figure 2:**
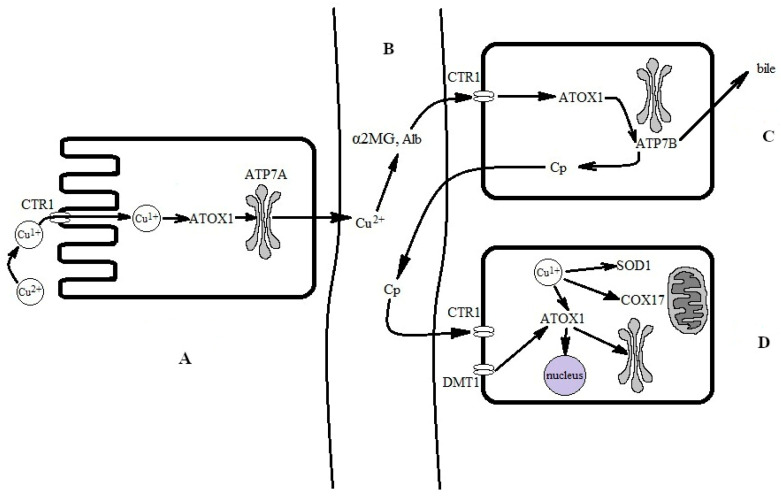
Simplified scheme for the conversion of food-derived copper into biovaluable copper. (**A**) Absorption of copper from the intestine. Copper is absorbed via enterocytes through CTR1. Transfer through CTR1 is coupled to a reduction to the copper(I) state. Copper(I) is bound by the ATOX1 chaperone, which passes it to ATP7A for excretion to the interstitial space and subsequent transfer in the bloodstream. (**B**) In extracellular fluid and the bloodstream, copper is oxidized to a copper(II) state and bound by serum albumin (SA) and α2-macroglobulin (α2MG). Copper(II), now bound to SA and α2MG, is carried to the liver via the portal vein and is then captured by CTR1 at the hepatocyte surface. Supposedly, it is reduced at the surface, but the reductase is not yet identified; presumably, copper(II) may be reduced by SA [97]. For simplicity, the DMT1 pathway and local copper distribution in the enterocyte are not shown. (**C**) In the hepatocyte, copper(I) is trafficked to the Golgi complex by ATOX1 and ATP7B, where it is inserted into newly synthesized Cp, which is secreted into the bloodstream. Local copper distribution in the hepatocyte is not shown. (**D**) The holo-Cp delivers copper to CTR1 on non-hepatocyte cells, which imports copper into the cells [98].

In adult mammals, when ingested with solid food or drink, copper dissociates from the proteins in acidic gastric conditions and associates in the copper(II) state with either amino acids (Cys, His, Asp, Met, Tyr, Gly, and Thr) or organic acids (gluconic, lactic, citric, and acetic acids) [95]. Two copper importers are located in the apical membrane of the enterocytes: divalent metal ion transporter 1 (DMT1) and channel-like high-affinity copper transporter 1 (CTR1) (Figure 2) [96].

Copper(II) ions that were imported into the cell via the DMT1 transporter can be converted to copper(I) in many reactions, most probably in reactions with major cellular thiols, such as glutathione or metallothioneins. Alternatively, they can be used in a copper(II) state via copper-dependent regulatory proteins [99]. CTR1 transfers copper ions through the membrane in the copper(I) state; therefore, extracellular copper(II) ions have to be reduced by reductases at the cell surface (Figure 2A) [100]. Inside the cell, copper(I) ions are bound by the apo-Cu-chaperone ATOX1, which carries copper(I) ions to the copper(I)-binding sites of ATP7A, a copper-transporting P-type ATPase that is mutated in Menkes disease [71,101]. ATP7A pumps the copper to exocytic vesicles, which excrete it to the extracellular space of the organism in a copper(II) state [102].

Once copper enters the bloodstream, it is rapidly bound to serum albumin (serum albumins in many species contain an N-terminal ATCUN motif, which has a high affinity to copper) [103] and to α2-macroglobulin (α2MG) [104]. The amounts of copper bound to serum albumin (serum protein concentration of ~70 mg/mL) and α2MG (serum protein concentration of 1.2 mg/mL [105]) are comparable (Figure 2B). Radioactively labeled copper is detected in both proteins almost immediately after the label injection [106]; these proteins carry all the absorbed copper ions to the liver (Figure 2C). The liver is the central organ of copper metabolism and enables the strict regulation of copper homeostasis in the whole body.

The albumin copper fraction enters the hepatocytes through CTR1 interaction with albumin, and the entry is coupled to its reduction to a copper(I) state [97,107]. CTR1 delivers copper(I) directly to the cytosol, while α2MG enters the endolysosomal compartment via endocytosis [108]. There, at low pH values, copper(II) is released and reduced to copper(I) by the metalloreductases of the STEAP (six-transmembrane epithelial antigen protein) family [109]; then, copper(I) is transferred from the endolysosomal compartment to cytosol via the low-affinity copper transporter, CTR2, which is homologous to CTR1 [110]. Thus, serum albumin and α2MG are components of the copper exchange pool for extracellular fluid, blood plasma, hepatocytes, and various organs (Figure 2C,D).

### 2.4. Copper Forms into Several Pools in the Hepatocytes, Which Interact with One Another

In the hepatocytes, absorbed copper is bound by the specialized cytosolic Cu(I)-chaperones, COX17, CCS, and ATOX1 (Figure 2C). COX17 delivers copper to the mitochondria for loading into cytochrome-*c*-oxidase (COX or complex IV), as well as to SOD1 in the intermembrane space. Several dedicated Cu(I)-chaperones (COX1, COX2, COX11, Sco1, and Sco2) insert copper ions into the active site of the assembling COX [74,111]. In addition to catalytic copper, there is a deposit of copper in the mitochondrial matrix. Copper is brought to the mitochondrial matrix via a phosphate carrier protein (the product of the SLC25A3 gene) and is stored in the complex with the copper ligand (CuL). CuL can store copper or mobilize it for the formation of cytosolic and mitochondrial cuproenzymes [112]. Therefore, mitochondria possess their own autonomous copper pool, which consists of both catalytic and deposited fractions.

CCS, another copper(I) chaperone, delivers copper to Cu,Zn-SOD1 and facilitates its insertion into the active site of the enzyme. The copper in SOD1 comprises the catalytic pool of the cytosol. A fraction of the copper in the cytosol is bound to metallothioneins 1 and 2; together with glutathione, these proteins provide a redox system for cuproenzyme-independent copper(I)↔copper(II) conversion. The metallothionein fraction can deposit copper in the cytosol; after deposition, this copper can be recruited for apo-SOD1 and other cell needs.

The third cytosolic Cu(I)-chaperone, ATOX1 (antioxidant 1), performs several functions. It delivers copper to the copper-transporting ATPases, carries copper to the cell nucleus, serves as a transcription factor, and can also function as an antioxidant against superoxide and hydrogen peroxide [77,113,114]. The delivery of copper by ATOX1 to the cytosolic metal-binding sizes of ATP7B, a P1-type ATPase, is its most widely studied function [115,116].

Mutations in the ATP7B gene are responsible for the development of Wilson’s disease (WD) [117], an inherited autosomal recessive monogenic inheritance disorder [118]. ATP7B is highly homologous to ATP7A, but it is mostly expressed in the cells of organs that synthesize and secrete Cp, a multicopper blue oxidase that is also the major blood serum cuproenzyme [119]. The liver, the lactating mammary gland, and certain brain regions (see more details below) are known examples of its expression [120,121,122]. ATP7B carries out two major functions [123]. First, it actively translocates copper to the lumen of the Golgi complex and delivers it to the active centers of maturing Cp, which is then secreted to the bloodstream [124].

Radiolabeled Cp appears in the bloodstream about 90 min after the delivery of radioactive copper via serum albumin or α2MG [106]. In humans, about 95% of serum copper is bound to Cp and is, thus, not dialyzable [125]. Cp transfers copper to the CTR1 of non-hepatocyte cells [98,126]. Thus, the CTR1→ ATOX1→ ATP7B→ Cp axis is a system that processes nutrient copper into Cp-associated copper; the latter is used by the cells of various organs. Consequently, Cp is a key participant in copper exchange and is the key marker of copper status [127]. The second function of ATP7B is the excretion of excess copper to bile. This portion of copper is then removed from the organism.

## 3. Proofs for the Existence of the Link between Copper Dyshomeostasis and the Risk of Parkinson’s Disease Development

Several facts exist that point to the link between inherited disorders related to copper metabolism and the risk of PD development. First, the neurological symptoms of PD patients overlap with the clinical symptoms of WD. The human *ATP7B* gene is located in chromosome 13 q14.3; it spans about 80 kb of genomic DNA and contains 21 exons. The mature transcript contains about 7.5 kb of genomic DNA and codes a 1411 aa protein [128]. As noted above, WD is caused by mutations in the ATP7B gene that impair the activity of this copper pump, blocking the hepatic copper routes to apo-Cp in the Golgi complex and to bile via excretion (Table S1 in the Supplementary Materials) [129]. Currently, more than 600 ATP7B pathogenic variants have been identified that are associated with WD. These include single-nucleotide missense mutations, nonsense mutations, insertions/deletions, and splice site mutations (OMIM# 606882; https://www.omim.org/entry/606882 access on 21 August 2023).

WD is typically caused by the compound heterozygosity of mutated ATP7B alleles. The alteration of the ATP7B product blocks the excretion of excess copper into bile. As a result, the excess copper is accumulated in the liver, brain, and other tissues. Conversely, copper deficiency in the Golgi complex of hepatocytes causes a decrease in holo-Cp levels, the copper/protein ratio in Cp, and the total copper content in blood serum (<20 mg Cp/100 mL and ~500 µg of copper/L in WD patients, versus about 35–38 mg of Cp/100 mL and ~1000 µg of copper/L in healthy individuals). Consequently, the availability of biovaluable copper for the various tissues and organs decreases. In approximately one-third of PD patients, the Cp content and copper concentration in blood serum correspond to those found in heterozygous carriers of mutations in the ATP7B gene (WD carriers) [130,131,132]. The similarity of neurological symptoms in WD and PD patients (Table S1 in the Supplementary Materials) as well as the high frequency of lowered copper status indexes in PD patients (meta-analysis; Figure S2 in the Supplementary Materials) allow us to suggest that many PD cases are heterozygote forms of WD [133].

This assumption is supported by several reported cases in which patients with a clinical presentation of typical PD had one mutant ATP7B allele. In one study, three out of five sisters from a family in Sardinia who were diagnosed with PD at the age of 70 (identified as very-late-onset major depression (DSM-IV, 4th ed) and parkinsonism) possessed a 15 bp deletion at the 5′-UTR region of a single allele in the ATP7B gene [134]. PCR analysis of the mutations in “classical” PD-related genes (α-synuclein, parkin, and LRRK2) yielded a negative result. Oxidase activity and the copper concentration in their blood serum, as well as urinary copper excretion or the hepatic markers, were within normal ranges in these patients. In the other two sisters of the family, no traits related to PD or deletions in the 5′-UTR region of ATP7B were observed.

Conversely, a female patient from Spain was diagnosed with young-onset Parkinson’s disease at the age of 38 [135]. Her serum copper concentration and ceruloplasmin level were low, and her urinary copper levels showed fluctuations. The genetic test for Wilson’s disease showed a compound heterozygosity at exon 6 and exon 8 of the ATP7B gene (both mutations were characteristic of the Spanish population). No mutations were found in Parkin or LRRK2. However, her sister showed the same ATP7B genotype but did not display any signs of PD. In a cohort of 103 patients from Germany with early-onset PD (aged 41 ± 6.8 years), a single patient was identified who had an ATP7B allele with a mutated exon 14, which resulted in a change to H1096Q in the nucleotide-binding domain (this mutation is detected in almost half of all Caucasian WD patients) [136].

A similar case exhibiting the early onset of clinical PD symptoms in a heterozygous H1096Q mutation carrier was described in a cohort from Poland [137]. In Russia, another study reported a patient with early-onset PD and a novel mutation that led to the C1079G substitution of a conserved cysteine residue in the ATP7B nucleotide-binding domain [138]. In the latter case, the holo-Cp concentration level was at first within a normal range; however, during 8 years of observation, the holo-Cp concentration significantly decreased, while the non-ceruloplasmin copper level increased. Conformation studies of the mutated domain via molecular dynamics indicated that the substitution resulted in a decreased affinity of the domain to ATP.

The PD phenotype can also develop in association with the post-transcriptional suppression of ATP7B gene expression. For example, serum miR-133b contains a 7 nt sequence that is complementary to a sequence found in the 3′-UTR region of ATP7B-mRNA, which might modulate ATP7B gene expression, according to TargetScan [139]. A low level of miR-133b expression was correlated with the ceruloplasmin levels found in patients with PD. Moreover, the downregulation of miR-133b in the midbrain of PD patients and in mice models of PD has been identified in previous studies [140,141].

Thus, low levels or the reduced activity of ATP7B, manifesting in a decrease in holo-Cp content in the circulation, may be considered a justified risk marker of PD development. However, not all PD patients have lowered holo-Cp concentrations, and only a small number of PD patients are heterozygous carriers of ATP7B mutations. Not all WD heterozygote patients develop PD symptoms, and there are no common clinical and biochemical phenotypes recorded in PD patients with the ATP7B gene mutation. While most heterozygous carriers of WD copper status indexes are correlated with gene dose and the Cp level comprises about 20 mg%, the concentration of copper in urine remains low, Kayser–Fleischer rings do not form, and no neurological symptoms are observed. In many PD patients, the co-segregation of the identified variants with the disease phenotype in the family has not been established because of a lack of family members/medical history.

However, the arguments offered above become less significant if the substantial heterogeneity and variability seen in WD clinical presentation are taken into account. Consequently, the diagnosis of Wilson’s disease remains challenging for physicians. WD has hepatic and/or neurological forms; WD patients range from mild to severe cases, and the age of onset varies from 2 to 60 years. Even if the WD genotype is identified, the major clinical and biochemical markers may be absent. On the contrary, ATP7B mutations may not be readily revealed, even if distinct WD symptoms are present. The latter situation may be explained by the limited range of genetic analysis: often, only the exon with the most frequent mutations is tested; in more accurate studies, the exons and exon/intron junctions are sequenced. The distant part of the ATP7B gene promoter, as well as the intron sequences, are still almost unexplored. The role of the 5′- and 3′-UTR of ATP7B-mRNA, along with the other participants of post-transcriptional regulation of this gene, are also poorly understood.

The second argument supporting the link between the risk of PD development and abnormalities in copper metabolism is the similarity between the neurological symptoms of PD and aceruloplasminemia (aCp) [142,143]. aCp is a rare monogenic autosomal recessive disease that develops due to mutations in the Cp gene. It is characterized by a triad of symptoms: retinal degeneration and blepharospasm, diabetes mellitus, and mild neurodegeneration [144]. Biochemically speaking, aCp manifests itself in the absence of Cp in the circulation (in aCp heterozygotes, the Cp content comprises 0.2–0.6 g/L, versus about 350 g/L in healthy individuals) [145]. The causes of holo-Cp deficiency in WD and aCp are different. In WD patients, the *Cp* gene is intact, and the Cp-mRNA directs the synthesis of a full-length Cp polypeptide to the endoplasmic reticulum; apo-Cp is delivered to the Golgi complex, where it normally accepts copper ions from ATP7B. The insertion of copper ions is cooperative and is coupled to the correct folding of the holo-Cp molecule [146]. In WD patients, there is a deficiency of biovaluable copper in the Golgi lumen; therefore, the holo-Cp content lowers and its concentration in the blood drops to 3–10 mg%, versus ~35 mg% in healthy individuals. Conversely, in aCp patients, there is no deficiency of copper in the Golgi lumen; however, the apo-Cp protein is missing or defective and cannot bind copper, so holo-Cp is absent from the circulation. As a result, both WD and aCp, which was initially described as a WD variant, are similar in terms of the presence of holo-Cp deficiency. Both disorders promote the development of PD-like symptoms and phenotypes. Thus, it has been shown that single-nucleotide polymorphism in the Cp gene, which results in lowered Cp production, is associated with the risk of development of the PD phenotype [139].

In addition to mutations and polymorphisms in the *Cp* gene, *Cp* gene activity can be suppressed post-transcriptionally. The binding of specific protein complexes, which are induced by ROS or interferon gamma, with cis-elements in the 3′-UTR of Cp mRNA can modulate its translation [147,148,149]. A similar phenotype may be caused by a disturbance in Cp-mRNA translation that is critical for holo-Cp level in the circulation [150]. Additionally, several microRNAs can reduce the rate of Cp-mRNA translation [151,152,153]. Thus, the decrease in holo-Cp caused by post-transcriptional gene silencing may be responsible for the onset of the PD phenotype.

In their analytical paper, Roy et al. reviewed the approaches that are used to explain apparent WD cases with no mutations in the ATP7B gene [154]. They considered mutations in the genes *CTR1* (*SLC31A1*), *ATOX1*, and *COMMD1* (formerly MURR1, associated with copper toxicosis in Bedlington terriers [155]) as potential modifiers of copper metabolism, which may result in the initiation of a WD- or PD-like phenotype. Therefore, an identified mutation in the ATOX1 gene, leading to G14S substitution, can disrupt protein–protein interactions between ATOX1 and the copper-binding domain 4 of ATP7B, according to the in silico data. This can disturb the transfer of copper to the Golgi lumen and may be responsible for the phenotypic variations seen in WD [156]. Recently, it has been shown that two mutations in the CTR1 gene in a homozygous state cause fatal copper deficiency, which also manifests as a low concentration of holo-Cp [157,158]. Hypothetically, the heterozygous state of these mutations can result in altered copper metabolism and may present a risk for PD development. Apparently, the COMMD1 gene should be excluded from the list: while mutations in COMMD1 cause the toxic accumulation of copper in the liver, kidneys, and brain (which resembles the WD phenotype), the level of holo-Cp in serum matches normal ranges or is even slightly elevated [159].

The listed examples support the hypothesis that a portion of idiopathic PD cases are provoked by copper dyshomeostasis, which develops due to mutations in those proteins that implement the delivery of nutrient copper to cuproenzymes and copper-dependent regulatory proteins. It suggests that a decrease in the level of holo-Cp is a critical factor in the risk of developing PD associated with copper dyshomeostasis. This is determined by the molecular functions of Cp and their role in the development of phenotypically similar neurodegenerative diseases.

## 4. Ceruloplasmin Is a Moonlighting Multicopper Blue Oxidase

Ceruloplasmin (Cp) is an oxidase cuproenzyme and a copper-binding protein found in vertebrates. In humans, the unique *Cp* gene maps to the chromosome region 3q23–q24 [160,161]. The *Cp* gene spans approximately 65 kbp and contains 20 exons; exon 20 codes the *C*-terminal transmembrane domain, which contains the site for the addition of a glycosyl phosphatidylinositol anchor (GPI anchor). The primary Cp transcript is processed into two isoforms of Cp-mRNA via alternative splicing, which code for secretory Cp and the membrane-anchored GPI-Cp [162,163,164]. There is also a Cp pseudogene, a 3′-terminal region of the processed *Cp* gene, which maps to chromosome 8 and was shown to be expressed in cultured human cells. The *N*-terminus of the putative pseudo-Cp is predicted to have a signal sequence for delivery to the mitochondria [165,166,167].

The regulation of the alternative splicing of the *Cp* primary transcript is tissue-specific; it depends on the physiological status of the cell and the period of ontogenetic development [122,168]. Therefore, the major fraction of secretory Cp is synthesized by the liver; it is also synthesized in lactating mammary glands and in the developing brains of newborns, the choroid plexus, and the spleen. GPI-Cp is synthesized in Sertoli cells, mature astrocytes, and neurons, as well as in the cells of the kidneys, lungs, adrenal glands, spleen, retina, the differentiating cells of mammary glands, white adipose tissue, and fetal tissue, but it is not synthesized in hepatocytes. In the liver, two molecular isoforms of mRNA-coding secretory Cp were revealed (3.7 kb and 4.2 kb), which are formed by the alternative cleavage and polyadenylation of 3′-UTR [160,162]. The different lengths of 3′-UTR suggest that the two mRNA forms differ in terms of the essential cis-elements for transcript-specific translational control.

Cp is synthesized on the polyribosomes associated with the membranes of the rough endoplasmic reticulum; it is directed there by the 19-residue signal peptide, which is removed co-translationally [169]. The mature translation products of Cp-mRNA and GPI-Cp-mRNA have an approximate molecular mass of 132 kDa. Four *N*-glycosylation sites have been identified experimentally, in which the glycoside chains are sialylated di- and triantennary [170]. Upon maturation, the protein binds six copper ions in three non-equivalent catalytic sites, along with two labile copper ions [120,171,172]. Both the soluble and membrane-anchored Cp isoforms belong to the vast family of blue multicopper oxidases. This family comprises multidomain proteins of diverse species, from bacteria to vertebrates, which use copper ions to catalyze various redox reactions [119,172,173]. In mammals, this family is represented by four members [119]: the two Cp isoforms and two single-span transmembrane proteins, hephaestin [174] and zyklopen [175]. They are very similar, both structurally and functionally. At the primary structure level, hephaestin and zyklopen demonstrated 50% and 46% identity to Cp, respectively, and hephaestin and zyklopen are 49% identical. Single-gene knockout studies in mice have shown that hephaestin and Cp play mutually compensatory roles in facilitating iron efflux [176].

These proteins have a similar arrangement of coordination spheres in the copper-binding site and also possess a high-affinity iron(II)-binding size, which is used to convert iron(II) to Fe(III); hence, they belong to the ferroxidase group. Dioxygen serves as an electron acceptor; it is bound in the multicopper site and is reduced completely to two water molecules [177]. The ferroxidase activity of Cp, GPI-Cp, hephaestin, and zyklopen is critical for the control of iron metabolism. These oxidases can also oxidize abiogenic aromatic amines (e.g., *p*-phenylenediamine and *o*-dianisidine). The loss of ferroxidase activity in any of these proteins results in abnormal iron accumulation, which leads to iron-related metabolic disorders, including neurodegenerative diseases [178,179].

In addition, Cp was shown to have other functions: it can defend against oxidative stress and possesses oxidase activity toward NO and the biogenic amines (serotonin, dopamine, adrenaline, and noradrenaline). Cp is an acute phase protein; its concentration in blood plasma rises several times in the event of inflammation, various infections, tumor growth (due to its role in neovascularization), pregnancy, etc. [119,180,181]. One of the key functions of blood serum Cp is its copper transportation function [98,126,182].

Cp can be attributed to moonlighting proteins by four criteria: a change in protein localization; a change in where or when a protein is expressed; post-translational modification; and a change in binding partners [183]. Therefore, there are two protein products of the unique *Cp* gene resulting from alternative splicing: a soluble extracellular form and a GPI-anchored form in the cell membrane. Secretory Cp is synthesized by mammary gland cells only during lactation [168]. It is also synthesized by the cells of neuronal tissue in the early stages of postnatal development (Table S6 in the Supplementary Materials) and it probably modulates the organization of neuronal tissue in ontogenesis [184]. In adult mammals, the GPI-Cp splice isoform is selectively expressed in astrocytes [185].

In newborns, the serum Cp expression level in the liver is low; however, after the switch of the copper metabolism to the adult type (see below), the liver becomes the main serum-Cp-producing organ [186]. Cp controls the trafficking of iron, both into and out of the cells. Apo-transferrin is the molecular partner of Cp in terms of iron importing: iron(III) is transferred after iron(II) uptake and oxidation by ceruloplasmin to the *C*-lobe of transferrin, in a protein–protein adduct [187]. In iron export, GPI-Cp works in tandem with ferroportin [188]. The copper transport function of Cp is mediated by its interaction with the extracellular domain of CTR1 [98,189]. As with most of the extracellular proteins, Cp is not subject to reversible covalent modifications. Its membership of the moonlighting proteins group makes Cp a strong candidate for involvement in the development of a group of diseases [190]. The data from the following meta-analysis support this assertion.

## 5. Updated Meta-Analysis on the Significance of the Link between Copper Status Indexes and the Risk of PD Development

In the first step of this study, the author M.N.K. searched the MEDLINE database (https://www.nlm.nih.gov/medline, NIH, USA) for the terms “copper”/“Cu” AND “Parkinson disease” on 17 July 2023. All articles written in English that were published before the search date were initially included in the analysis. No search restrictions regarding analytic methods or experimental approaches (i.e., in vitro or in vivo methods) were applied. In total, 654 unique articles were prescreened manually by two authors (M.N.K. and E.Y.I.), according to predefined inclusion criteria (quantitative measurements of copper in human blood from PD patients); 622 records were excluded, and 32 articles were further assessed for their eligibility. Fourteen articles were selected for meta-analysis (Figure S2 in the Supplementary Materials).

In the second step, the author M.N.K. searched MEDLINE for the terms “ceruloplasmin”/“Cp” AND “Parkinson disease” in July 2023, in the same way. In total, 134 unique articles were prescreened by two authors (M.N.K. and E.Y.I.) according to the predefined inclusion criteria (quantitative measurements of ceruloplasmin in human blood from PD patients); a total of 112 records were excluded, and 22 articles were further assessed for their eligibility. Eleven articles were selected for meta-analysis (Figure S2 in the Supplementary Materials). The characteristics of each study (the number of samples and mean copper or ceruloplasmin levels, with standard deviations) were extracted for analysis. The primary outcome was the differences in copper/ceruloplasmin concentrations between PD and control tissues.

**Figure 3:**
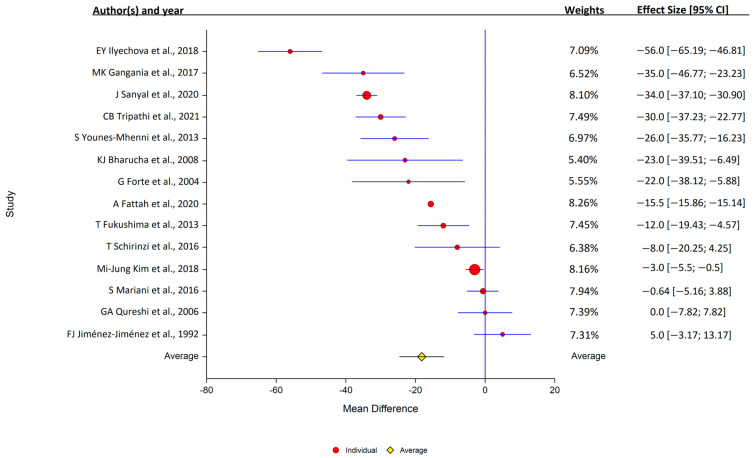
Forest plots of the analysis of copper in human serum. Circular marker indicates the effect size and statistical weight of the study; horizontal lines indicate 95% CI values. The diamond-shaped data marker represents the overall effect size and 95% CI. The vertical line shows the line of no effect (d = 0).

Of the 14 studies that assessed serum copper levels, 11 found that the levels were lower in PD, while 3 found that they were similar in the PD and control samples. Overall, serum copper levels were lower in PD patients (14 studies: PD, 1377 samples; control, 1283 samples); study heterogeneity was high (I^2^ = 98.3%; Q, 419; *p* < 0.001) (Figure 3).

**Figure 4:**
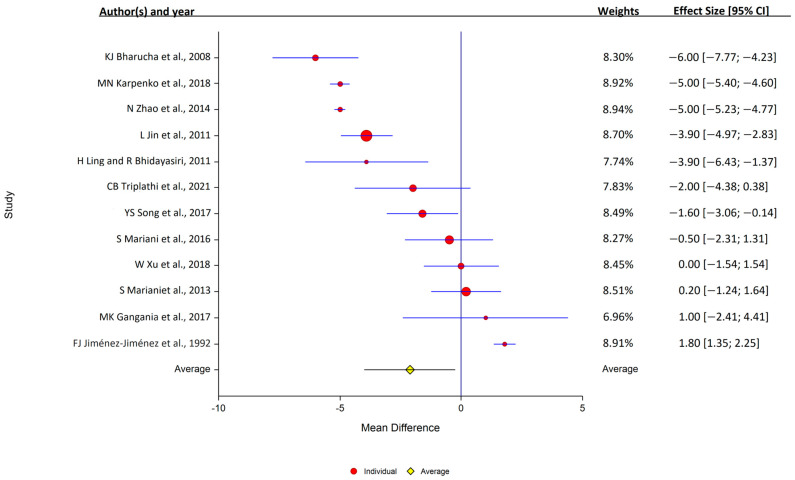
Forest plots for the analysis of ceruloplasmin in human serum. Circular marker indicates the effect size and statistical weight of the study; horizontal lines indicate 95% CIs. The diamond-shaped data marker represents the overall effect size and 95% CI. The vertical line shows the line of no effect (d = 0).

Of the 12 studies that assessed serum ceruloplasmin, 7 reported that levels were lower in PD, and 5 reported that the levels were similar in the PD and control samples. Overall, serum ceruloplasmin levels were lower in PD patients (12 studies: PD, 1004 samples; control, 708 samples); study heterogeneity was high (I^2^ = 98.9%; Q, 847; *p* < 0.001) (Figure 4).

A list of the references included in the meta-analysis is given in Figure S2 of the Supplementary Materials.

Therefore, the meta-analysis emphasized that the concerted measurement of all indexes of copper status is required to establish a connection between PD and disorders in copper homeostasis, including measuring the concentrations of copper weakly associated with the Cp molecule [138].

## 6. Non-Ceruloplasmin Copper as a Perspective Marker for Neurodegeneration

Recently, an increasing number of papers have been demonstrating a positive correlation between the risk of neurodegenerative disease development and the level of copper fraction in blood plasma, which is alternately denoted as “free” copper [191,192], “extractable copper” [193], “exchangeable copper” (ExCu) [194,195], or “non-ceruloplasmin copper” (NCC) [192,196,197,198,199,200,201,202,203].

Conventionally, Cp copper is the fraction associated with immunoreactive Cp; in healthy humans, it comprises about 90–95% of total serum copper [125]. Transit copper, which is associated with serum albumin and α2MG, comprises a major part of the remaining fraction (about 10% of total copper). The residual minor fraction of copper that is not associated with serum proteins is measured via ultrafiltration, titration with luminescent chelators, or specific carriers. It can be also measured via the subtraction of protein-associated copper concentration from the total plasma serum copper concentration. Greatly increased NCC concentrations were reported in the late stages of WD and in some forms of Alzheimer’s disease (AD) [204]. It has been shown that the NCC level correlates positively with the C-reactive protein level, i.e., it is connected to the inflammation processes. However, an increased NCC fraction was not observed in PD patients [205].

In *ATP7B*−/− mice, which represent an experimental phenocopy of WD, a small copper carrier (SCC) has been identified in the urine. It is a ~1 kDa non-proteinaceous molecule, the concentration of which is correlated with the development of the WD phenotype. In mice lacking a functional *CTR1* (*SLC31A1*) gene, it was observed that SCC not only provides copper excretion from the liver but can also perform copper importing into the cells [206]. However, while an increase in SCC level was observed in *ATP7B*−/− mice and dogs, as well as in WD patients, it was shown later that SCC is secreted by cultured cells of different origins, and that [^67^Cu]-SCC is the copper donor for these cells [207]. In the cytosol of the liver of rats that chronically received silver ions with food, a low-molecular-weight agent appeared that could facilitate copper(I)↔copper(II) transition [208]. Although these studies are currently fragmented and not ordered, we think that NCC may become a valuable diagnostic marker of copper dyshomeostasis, with an increased risk of neurodegeneration.

## 7. Disorders in the Brain’s Copper Metabolism Associated with Parkinson’s Disease

The data that definitely show the existence of a link between low Cp and Cp-associated copper levels, and the risk of PD development and the risk of severe PD in such patients, refer to copper indexes in the peripheral blood (Figure S2, meta-analysis in the Supplementary Materials). At the same time, in PD patients, indexes of copper metabolism in the brain may differ from those of the blood. Unfortunately, there is very little coherent information on copper homeostasis in the brain, let alone its changes in the context of PD.

It has been shown that nutrient-based or genetically determined copper deficiency caused a decrease in the brain-specific and ubiquitous cuproenzymes in the brain. As a result, severe dysfunctions of the central nervous system may develop, including those with the PD phenotype [6,209,210,211,212,213,214]. The manifestation of this deficiency is seen in the alteration of copper content in brain regions, including the SN. Therefore, in healthy people, copper concentration in this brain region is higher than that in the adjacent regions. Conversely, in PD patients, it is lowered by almost one-third [215]. At the same time, copper redistribution in the cell is observed; it concentrates in the insoluble fraction including the Lewy bodies, in SOD1 and α-synuclein aggregates, and in a minor (by mass) fraction of small insoluble proteinaceous deposits [216]. Generally, copper contents in the brains of PD patients are lower than normal physiological ranges, unlike WD patients [217].

Investigation of the cerebrospinal fluid (CSF) is valuable for understanding the mechanism of PD development. Changes in the structure of Cp from CSF, which were not present in serum Cp, were described in PD patients [218,219]. These changes include the oxidation and deamidation of the Cp molecule. Cp oxidation ultimately leads to the loss of its ferroxidase activity and iron accumulation in the cells [218,220,221]. The deamidation results in the Cp’s ability to bind to integrin, an important component of CNS signaling [222,223]. The binding of modified Cp triggers intracellular signaling on the choroid plexus epithelial cells, changing cell functioning, which might contribute to the pathological mechanism [224,225]. These data attract attention to the choroid plexus as the main CSF-producing organ and the major copper homeostatic site in the brain; the malfunctioning of the copper metabolism may be responsible for the development of the PD phenotype.

In mammals, the liver and the brain are the leading organs according to copper content in the body. Together, these organs account for about 30% of total body copper, while comprising less than 1/50 of the total body mass. Average copper concentrations in the brain and in the liver are similar [226]. In brain cells, as well as in hepatocytes, copper is mostly contained in the nuclei, mitochondria, and cytosol [227,228,229]. Both organs display a high level of expression of the ubiquitous cuproenzymes, COX and SOD1. Specific cuproenzymes are also expressed in the brain; they are mostly responsible for the synthesis, activation, and catabolism of neuromediators (Table 2).

However, unlike the almost uniform distribution of copper in the liver [241], copper is unevenly distributed across the regions of the brain; its distribution is not even uniform within the regions [242,243]. If liver copper may be viewed as a transitional dynamic pool, copper in the brain is more stationary. Copper metabolism in both organs changes in ontogenesis; it is then classified into embryonal-type copper metabolism (ETCM) and adult-type copper metabolism (ATCM), which have organ-specific traits (Table S2 in the Supplementary Materials).

In the liver of newborns, expression of the *Cp* gene is low; Cp and copper concentrations in blood serum comprise about 25–33% of the normal adult levels. The copper-to-Cp ratio is about 7 ions per single molecule; 1–2 ions can be extracted by Chelex-100, a Cu(II)-specific chelating resin (for loosely bound copper atoms, see Table S3 in the Supplementary Materials). ATP7B is not expressed in the liver in ETCM. The sole source of copper at this stage is milk Cp. The synthesis of milk Cp by lactating mammary glands is tightly regulated at the transcription level, so that the newborn receives a constant quantity of copper per body mass [168,244]. As the pH values in the stomach and duodenum of newborns are close to neutral, milk Cp is not degraded or stripped of its copper ions; instead, it is transferred via transcytosis to the bloodstream, together with its copper load [182].

The mechanism of copper delivery to the brain in ontogenesis is not sufficiently studied. In early studies, the distribution of radiolabeled copper from intraperitoneal [^67^Cu]Cl_2_ injection in adult rats was assessed. It was shown that in the early stages of the experiment, radioactive copper did not enter the brain. The label was rapidly detected in the liver, while in the bloodstream, [^67^Cu] appeared 1.5 h after the injection, as part of newly synthesized [^67^Cu]Cp. It was only after the level of labeled Cp started to decline that the label could be detected in the brain (3 h post-injection). The level of [^67^Cu] that entered the brain then persisted and did not decline [245]. In an alternative approach, radiolabeled [^3^H]Cp was injected intravenously into rats and the distribution of the [^3^H]Cp peptide part was monitored. Labeled Cp molecules were not detected in the brain [246]. These data suggest that serum Cp takes part in copper delivery to brain tissues, but its polypeptide does not cross the blood–brain barrier. Later, it was shown that ”free” [^64^Cu] copper ions injected into the brain via perfusion were more effectively absorbed by the choroid plexus cells than copper from [^64^Cu]albumin or [^64^Cu]Cp; however, copper delivered via [^64^Cu]Cp was transported to the brain parenchyma cells [247,248]. It should be noted that in these reconstruction experiments, liver copper metabolism was excluded; therefore, the amount of copper absorbed from [^64^Cu]Cl_2_ could have been overestimated.

In newborns, copper is actively transported to the brain; its concentration in the brain increases progressively up to 15–20 days after birth [249] (Table S4 in the Supplementary Materials). The polypeptide part of milk Cp does not cross the blood–brain barrier, although the latter has relatively high permeability at this stage of development [250]. Then, the copper transport rate decreases, while the copper concentration remains relatively constant [249] (Table S4). Ontogenetic variations of copper concentration in the choroid plexus are remarkable, and are very similar to those in the liver (Table S2 in the Supplementary Materials). In the cells of the choroid plexus, copper is accumulated during the first days after birth, and then its concentration decreases abruptly. Here, the expression level of both Cp-mRNA splice isoforms is the highest among other brain regions (Table S5 in the Supplementary Materials), and both ATP7A and ATP7B are expressed (they are typically co-expressed with GPI-Cp and secretory Cp, respectively) [247,251].

The gene of the CTR1 transporter, which is responsible for copper import and provides copper for Cp synthesis, displays the highest expression level in the choroid plexus (Figure S6 in the Supplementary Materials). Throughout life, the Cp level in CSF remains very low and does not correlate to Cp concentration in the circulation (Tables S3 and S6 in the Supplementary Materials). All copper in the CSF is precipitated by antibodies with Cp, i.e., the non-ceruloplasmin copper content is negligible. According to estimations, nine to ten copper ions are bound to the Cp molecule, and up to four can be removed with Chelex resin (Tables S3 and S6). These are evidence for the remarkably high content of labile non-dialysable copper in CSF Cp. It is highly likely that CSF Cp plays an important role in the delivery and excretion of copper to and from the cells of the nervous tissue and provides copper homeostasis in the brain. Possibly, Cp also takes part in neuronal differentiation and the formation of brain structures [252,253,254].

This consideration is supported by the data obtained from *ATP7B*−/− mice with the systemic deletion of the ATP7B gene. The loss of ATP7B caused a disturbance in the copper metabolism in the choroid plexus and copper and iron imbalance in the neurons (in particular, it caused copper deficiency in the neurons), which was aggravated with age [255]. Information about copper metabolism in the brain remains scarce; it does not allow us to picture a map of copper transfer in the brain between neuronal cells, CSF, and extracellular space. However, it allows us to outline a draft and to speculate that Cp, originating from the cells of the choroid plexus and circulating in the CSF, plays a central role in these events. Due to this finding, CMS Cp could possibly be the most valuable marker of copper-associated PD.

## 8. Conclusions

The cloning of the genes that are responsible for the development of Menkes disease and WD, achieved in 1993, may be considered as the starting point for systematic intensive research of copper metabolism [256,257,258,259,260]. The main achievement in this area is the identification of a special group of “copper-associated diseases”, which comprise oncological [261], cardiovascular [262], and metabolic disorders [263], as well as neurodegenerative diseases caused by inherited abnormalities in copper metabolism [217,264,265,266] and/or acquired copper homeostasis misbalance. PD is also attributed to this group [41,267].

In the final part of the presented article, which focuses on the role of copper imbalance in the risk of developing PD, we highlight its major points.

First, although copper is a transition metal and can catalyze the formation of ROS, neither increased environmental concentrations nor high dietary levels are associated with a risk of developing PD. This is explained by the fact that mammals have several levels of mechanisms for maintaining the homeostasis of essential and simultaneously toxic copper ions.

Second, the risk of developing a copper imbalance is associated with inborn errors in copper metabolism. These can have diverse manifestations: a decrease in holo-Cp level, a lowered copper load of the Cp molecules, or a decrease in the labile copper ion count per Cp molecule. This disrupts the functions of both molecular forms of Cp: ferroxidase and the copper transporter. As a result, copper deficiency develops in the cells, along with a simultaneous increase in iron concentration. Iron accumulation is an intrinsic feature of the degenerating brain regions in Parkinson’s disease. Therefore, cells with a function that is associated with cuproenzymes, such as SN cells, will die. In addition, neurodegeneration in the SN may be the result of a strong redox pair, formed by iron itself and dopamine.

Third, we explain the dissonance between the small number of identified genetic cases of PD, which are associated with copper imbalance and the change in copper status in about one-third of patients with PD (Tables S7 and S8 in the Supplementary Materials; [131]) via the insufficient study of the expression of the corresponding genes in patients with PD. Progress can be made with the obligatory measurement of the markers of copper status in terms of making a PD diagnosis.

Fourth, multiple pathways leading to the disruption of copper homeostasis may be responsible for cases in which serum copper status indexes are not impaired; however, an imbalance in copper in the CSF is observed. Unfortunately, very little research has been devoted to this issue.

Fifth, if the multiple pathways of genes responsible for holo-Cp formation and their multilevel regulation are considered, it may be supposed that holo-Cp and copper ions, which are associated with it, may be valuable markers for PD. Therefore, they can perhaps be used as prognostic markers for various subtypes of PD, similarly to the use of the non-ceruloplasmin copper level in the discrimination of clinical subtypes of AD [204].

Mammalian models are mainly used to study the molecular genetic mechanisms of PD development. However, their genetic complexity significantly complicates our understanding of primary disorders. Perhaps, progress in studying the role of copper imbalance in the PD phenotype development will be facilitated by studies on the C. elegans model, which is convenient for studying the role of copper dyshomeostasis because, like mammals, they express the CTR1→ATP7B/A→ATOX1→Cp axis from worm’s orthologues [268,269,270].

## Figures and Tables

**Table 2:** Function of ubiquitous and tissue-specific cuproenzymes in the brain.

Cuproenzyme	Localization in Brain	Biological Function	Relation between Abnormal Expression/Function and PD	Ref.
SOD1	ubiquitous, cytosol, mitochondrial intermembrane space	protection from oxidative stress via the conversion of O_2_^•−^ to O_2_ and H_2_O_2_ by disproportionation	in cases of copper misbalance, SOD1 misfolds and aggregates that result in the PD-phenotype developing	[230]
COX	ubiquitous, mitochondrial inner membrane	complex IV in ETC catalyzes the transfer of electrons from cytochrome *c* to O_2_	defects in ATP production and OXPHOS-independent functions of ETC proteins play an important role in PD pathogenesis	[231]
SOD3	ubiquitous, extracellular space, associated with matrix components	controls vascular tone and reactivity in the brain through the regulation of equilibrium between O_2_^•−^ and NO	polymorphism of the SOD3 gene is associated with PD risk	[232,233,234,235]
PAM	anterior pituitary secretory granules	catalyzes the conversion of glycine amides to amides and glyoxylate		[236,237]
DBH	secretory vesicles of central and peripheral nervous system [10,11]	catalyzes the conversion of dopamine to norepinephrine and to epinephrine	mutations in the DBH gene are associated with human norepinephrine deficiency	[238,239]
Tyrosinase	barely detectable, levels are not exactly defined	catalyzes the production of melanin from tyrosine via oxidation	reduced level of neuromelanin in SN	[6]
AOC3, or CAO3	not clearly established	catalyzes the oxidation of primary amines to aldehydes, with the release of NH_3_ and H_2_O_2_	not established precisely	[240]

Remarks: SOD1: cytosolic Cu/Zn-superoxide dismutase; SOD3: extracellular Cu/Zn-superoxide dismutase; COX: cytochrome-*c*-oxidase; PAM: peptidyl glycine alpha-amidating monooxygenase; DBH: dopamine-beta-hydroxylase; O_2_^•−^: superoxide radical; ETC: electron transport chain; AOC3—Amine oxidase (copper-containing)/vascular adhesion protein 1 (AOC3/VAP1).

## Supplementary Materials

The following supporting information can be downloaded at: https://www.mdpi.com/article/10.3390/antiox12091654/s1, Table S1. Features of neurological symptoms in WD and PD patients. Figure S1. Flow chart of the review process for analysis of human serum copper. Figure S2. Flow chart of the review process for analysis of human serum ceruloplasmin. Table S2. The peculiarities of the copper metabolism during development in the rat liver and brain. Table S3. The changes in copper and ceruloplasmin concentration in serum blood and CSF during development. Table S4. Redistribution of copper in the brain regions of rats during postnatal development. Table S5. Relative content (mRNA/actin mRNA) of mature transcription products of the Cp and CTR1 genes in the brain regions of 10- and 120-day-old rats. Table S6. Copper concentration in the CSF bound with ceruloplasmin. Table S7. Demographic and clinical characteristics of the PD patients and healthy volunteers. Table S8. Copper status indexes in PD patients (n = 50) and healthy volunteers (n = 50). Refs. [271,272,273,274,275,276,277,278,279,280,281,282,283,284,285,286,287,288,289,290] are cited in the Supplementary Materials file.
