# Supplementary material for: Abnormalities in Copper Status Associated with an Elevated Risk of Parkinson’s Phenotype Development
Source: Antioxidants (Basel). 2023 Aug 22;12(9):1654. doi: 10.3390/antiox12091654 (PMC10525645; doi:10.3390/antiox12091654)
Supplement: Supplementary file 1 [file antioxidants-12-01654-s001.zip › antioxidants-2480860-supplementary.pdf]

antioxidants

MDPI

Review

Abnormalities in Copper Status Associated with an Elevated Risk of Parkinson's Phenotype Development

Marina N. Karpenko [1,2], Zamira M. Muruzheva [1,3], Ekaterina Yu. Ilyechova [2,4,5,*], Polina S. Babich [6] and Ludmila V. Puchkova [2,4,5]

[1] I.P. Pavlov Department of Physiology, Research Institute of Experimental Medicine, 197376 St. Petersburg, Russia; mnkarpenko@mail.ru (M.N.K.); zamira.muruzheva@mail.ru (Z.M.M.)
[2] Institute of Biomedical Systems and Biotechnology, Peter the Great St. Petersburg Polytechnic University, 195251 St. Petersburg, Russia; puchkovalv@yandex.ru
[3] State Budgetary Institution of Health Care "Leningrad Regional Clinical Hospital", 194291 St. Petersburg, Russia
[4] Research Center of Advanced Functional Materials and Laser Communication Systems, ADTS Institute, ITMO University, 197101 St. Petersburg, Russia
[5] Department of Molecular Genetics, Research Institute of Experimental Medicine, 197376 St. Petersburg, Russia
[6] Department of Zoology and Genetics, Faculty of Biology, Herzen State Pedagogical University of Russia, 191186 St. Petersburg, Russia; babich.polina@gmail.com
* Correspondence: ilichevaey@itmo.ru; Tel.: +7-921-760-5274

Citation: Karpenko, M.N.; Muruzheva, Z.M.; Ilyechova, E.Y.; Babich, P.S.; Puchkova, L.V. Abnormalities in Copper Status Associated with an Elevated Risk of Parkinson's Phenotype Development. *Antioxidants* **2023**, *12*, x. https://doi.org/10.3390/antiox12091654

Academic Editor: Alessandra Napolitano

Received: 15 June 2023
Revised: 10 August 2023
Accepted: 16 August 2023
Published: 22 August 2023

Copyright: © 2023 by the authors. Submitted for possible open access publication under the terms and conditions of the Creative Commons Attribution (CC BY) license (https://creativecommons.org/licenses/by/4.0/).

Table S1. Features of neurological symptoms in WD and PD patients.

Clinical features	Wilson's Disease	Parkinson's disease
Rigidity	+	+
Tremor	+	+
Bradykinesia	+	+
Dystonia	+	+
Postural instability	+	+
Postural deformities	-	+
Myoclonus	+	-
Cerebellar dysfunction	+	-
Epileptic seizures	+	-
Dysarthria	+	+
Dysphagia	+	+
Olfactory dysfunction	-	+
Cognitive impairment	+	+
Depression	+	+
Acute psychosis	+	-
Autonomic dysfunction	+	+

S2. Meta-analysis

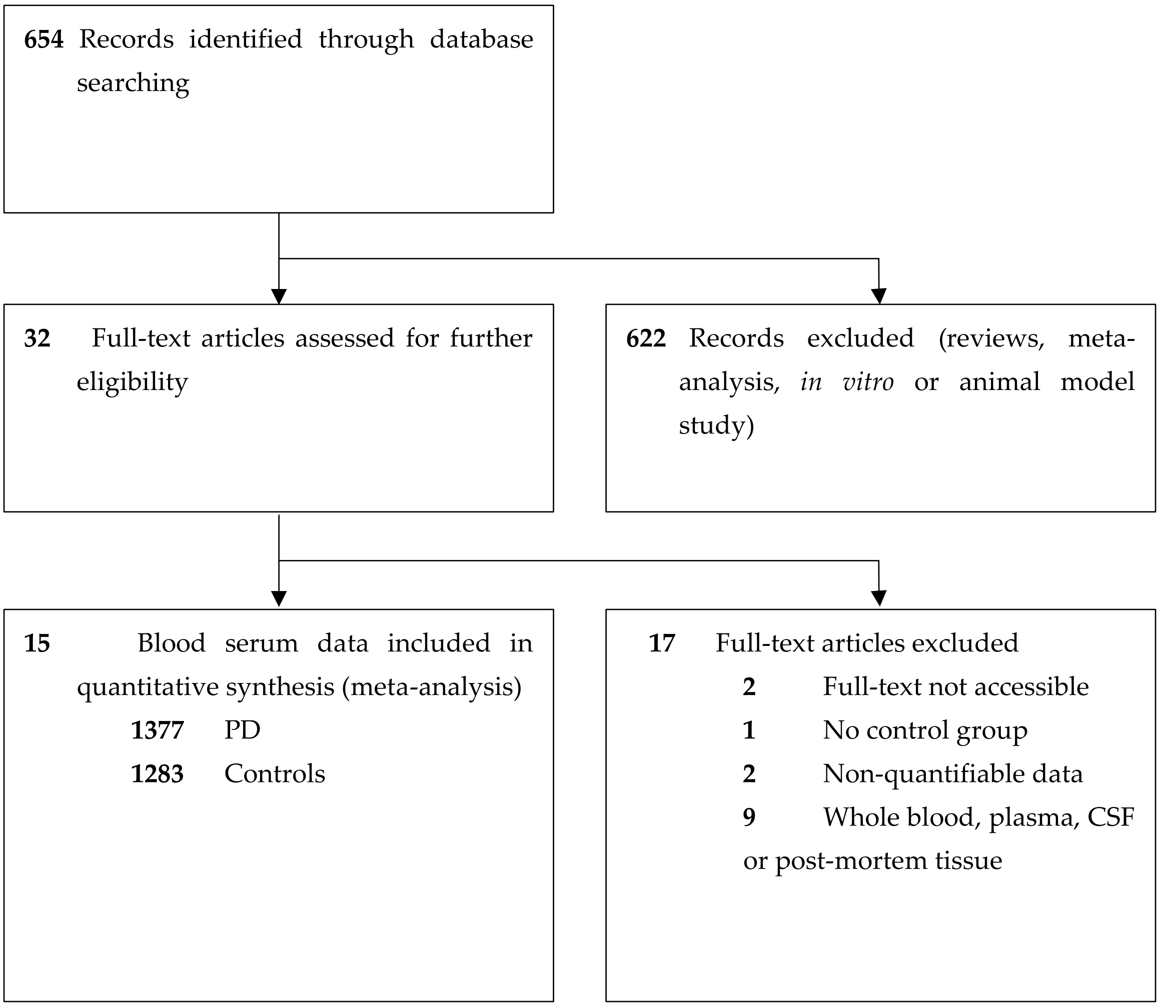

Figure S1. Flow chart of the review process for analysis of human serum copper. Data on sample size, mean (SD/SEM) copper concentration, and P values were extracted as primary outcomes [131, 271–282].

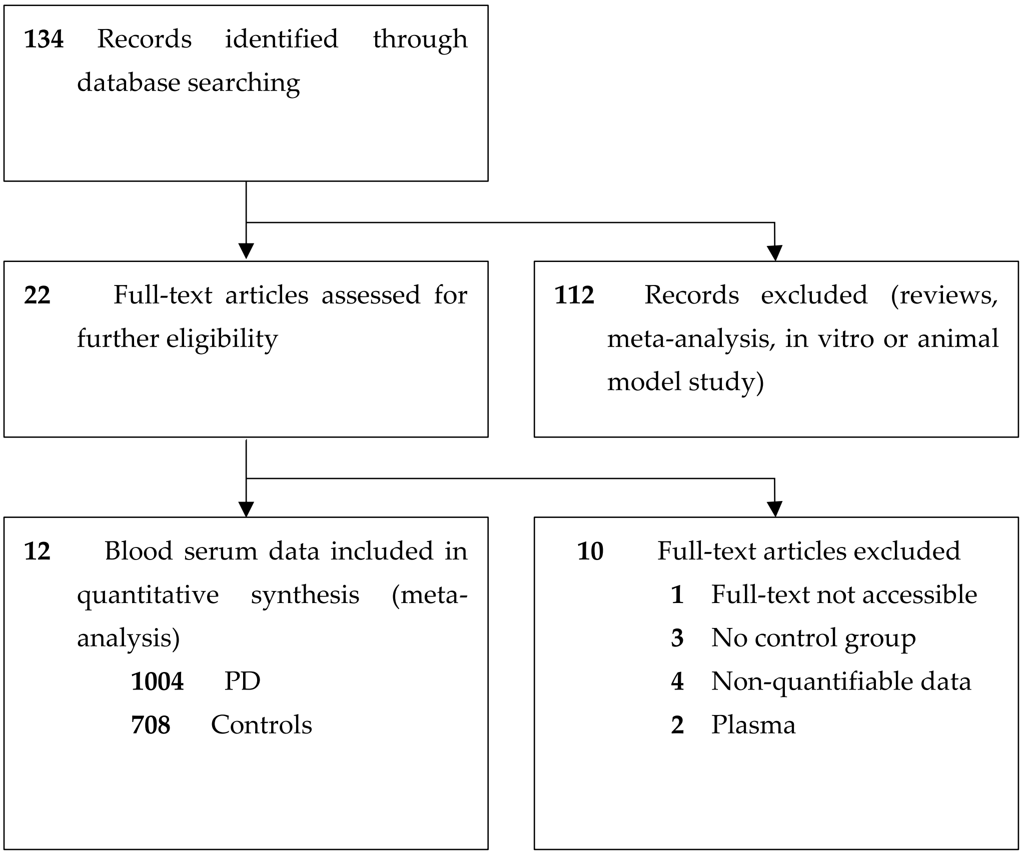

Figure S2. Flow chart of the review process for analysis of human serum ceruloplasmin. Data on sample size, mean (SD/SEM) ceruloplasmin concentration, and *P*-values were extracted as primary outcomes. Effect sizes were calculated as standardized mean differences of copper / ceruloplasmin concentrations between patients with PD and healthy controls and converted to the Hedges *g* statistic. We chose a random effects model for the meta-analysis. Between-study heterogeneity was assessed using the Cochrane *Q* test and I^2 statistic. Statistical difference for the Cochrane *Q* test was set at $P < 0.10$ [132, 139, 271, 274, 282–287].

Table S2. The peculiarities of the copper metabolism during development in the rat liver and brain.

SIGNS	LIVER		BRAIN	
	ETCM	ATCM	ETCM	ATCM
Start/switch	Starts in fetus and switches at 13th day of life [1]	Starts at 14th day of life [1]	Lasts up to 15-20 days of life [2, Tab. S3]	No drastic changes [2, Tab. S3]
Cp gene activity	+ [1]	+++ [1]	+++ [Tab. S7]	+++ [Tab. S7]
ATP7B gene expression	No expression	High expression	++	+
ATP7A gene expression	+	No expression	+	++
Cp, g/L, serum	~100 [1]	~325 [1]	n.a.	n.a.
GPI-CP	None	None	+ [Tab S7]	+++ [Tab S7]
[Cu], μg/g wet weight in liver or whole brain tissue	>500 [1]	~6 [1]	P0 ~1 ug/g; P14 ~2.5 ug/g [2, tab. S3]	P80 ~ 3 ug/g [2, tab. S3]
MT1 gene expression	+++	+	Not detected [3]	++ [4]
[Cu], μg/L in serum	~300	~950	n.a.-	n.a.-
[Cu], μg/L in CSF	n.a.	n.a.	~3 [Tab. S4]	~3 [Tab. S4]
Copper present in urine	+++	Not detected	n.a.	n.a.

Note: ETMC: embryonic type of copper metabolism; ATCM: adult type of copper metabolism; (+): low expression level; (+++): high expression level. P0, P14: day of life after birth; MT: metallothionein; n.a.: parameter not applicable to the organ [186, 249, 288–290].

Table S3. The changes in copper and ceruloplasmin concentration in serum blood and CSF during development*.

Age, days	Blood Serum			Cerebrospinal fluid (CSF)		
	[Cu], ug/L	Cp, g/L**	Cu atoms per one Cp molecule	[Cu], ug/L	Cp, g/L**	Cu atoms per one Cp molecule
6	200	59	~7	~15	~3	~10
14	240	80.6	~6.2	~15	~3	~10
160***	1600	512	~4.2	~15	~3	~10

Note: *The data were obtained using samples from the same rats; 7 µl of CSF or serum blood from three newborn rats was pooled. Two mixed CSF or serum samples were prepared. **Cp protein concentration was measured by rocket immunoelectrophoresis, the electrophoretic pure rat ceruloplasmin preparate was used as a standard. Immune zones were stained with *o*-dianisidine. ***Blood and CSF were taken from rats on the 8th day lactation. Copper concentration was determined by FAAS.

Table S4. Redistribution of copper in the brain regions of rats during postnatal development.

Brain department	Copper concentration, ng/mg total protein; age, days			
	P5	P10	P20	P120
Cortex	21.6±6.1	25.9±1.5	32.7±2.6*	24.92±3.01
Cerebellum	17.5±1.1	25.5±23.7	23.7±1.2*	32.34±3.56
Hippocampus	24.3±0.7	21.0±0.5	35.2±3.5*	32.82±8.48
Amygdala	10.5±1.2	32.0±8.1	30.7±11.3*	31.75±0.51
Hypothalamus	47.2±3.3	31.4±2.4	48.1±27.9	43.5±3.52
Pituitary gland	91.9±8.5	96.3±17.9	103.4±7.8	55.6±0.81
Choroids plexus	42.4±7.7	134.4±4.2*	100.6±7.9	2.46±0.26

Table S5. Relative content (mRNA/actin mRNA) of mature transcription products of the Cp and CTR1 genes in the brain regions of 10- and 120-day-old rats.

Brain region	Soluble Cp mRNA		GPI-Cp mRNA		CTR1 mRNA	
	**P10**	**P120**	**P10**	**P120**	**P10**	**P120**
cortex	0.35±0.013	0.06	0.05	0.1±0.05	0.91±0.01	0.8±0.0
cerebellum	0.15±0.003	0.13±0.007	0.05	0.21±0.019	0.93±0.07	0.75±0.03
hippocampus	0.21±0.017	0.06	0.09±0.003	0.1±0.001	0.61±0.04	0.83±0.03
amygdala	0.20±0.032	0.23±0.017	ND	ND	0.50±0.03	0.75±0.02
hypothalamus	0.25±0.019	0.25±0.07	0.16±0.007	0.54±0.063	0.45±0.01	0.75±0.01
pituitary gland	0.25±0.011	0.31±0.018	ND	0.37±0.015	0.6±0.003	0.59±0.02
choroid plexus	0.48±0.039	0.82±0.063	0.77±0.051	1.63±0.043	0.65±0.02	1.85±0.02

Note: ND – below detection threshold; P10 and P120: day of life after birth.

Table S6. Copper concentration in the CSF bound with ceruloplasmin.

CSF	Cu, µg/L	**Cp, g/L	Copper atoms per one Cp molecule
Without treatment	13.77±0.874	3	9
After Cp precipitation ***	0.53±0.094	Not detected	Not determined
After Chelex-100 treatment	8.87±0.697	3	5.7

* All measurements were performed in the same specimens, each specimen contained equal volumes of CSF taken from three rats; in total, three specimens of CSF (nine adult rats) were assayed. *** Variability in Cp concentration in specimens did not exceed 5%.

Table S7. Demographic and clinical characteristics of the PD patients and healthy volunteers.

Variable	PD patients (n = 50)	Healthy (n = 50)
Sex (Male/Female). n (%)	21/29 (42/58)	24/26 (48/52)
Age, years	64 (57;72)	60 (55;74)
Age of onset, years	55 (51;66)	n.a.
H&Y score	2.5 (1.5;2.5)	n.a.
UPDRS part III (motor part) score	28 (26;29)	n.a.
NMS PD score	14 (8;27)	n.a.
FAB score	16 (15;17)	n.a.
HADS «D» score	8 (6;10)	n.a.
HADS «A» score	7(4;9)	n.a.
S and E ADL score	80 (70;90)	n.a.
BDI score	18 (14;20)	n.a.
PIGD score	2(1;5)	n.a.
MMSE score	27(26;29)	n.a.

Notes: H&Y—Hoehn and Yahr stage; UPDRS—Unified PD Rating Scale; NMS PD—non-motor symptoms scale for PD; FAB—Frontal Assessment Battery; HADS—Hospital Anxiety and Depression Scale; SE-ADL—Schwab and England Activities of Daily Living Scale; BDI—Beck's Depression Inventor; MMSE—Mini-Mental State Examination; PIGD—Postural Instability and Gait Disorder. Data are represented as n (%) or median (IQR).

Table S8. Copper status indexes in PD patients (n = 50) and healthy volunteers (n = 50).

Parkinson's disease patients				Healthy			
N	[Cu], µg/L	[Cp], g/L	Cu atoms per Cp molecule	N	[Cu], µg/L	[Cp], g/L	Cu atoms per Cp molecule
1	629	0.32	4.0	1	1057	0.29	7.4
2	530	0.26	4.1	2	1322	0.49	5.5
3	751	0.34	4.5	3	738	0.23	6.5
4	421	0.18	4.8	4	1252	0.35	7.3
5	614	0.4	3.1	5	1514	0.42	7.3
6	904	0.39	4.7	6	1450	0.46	6.4
7	540	0.29	3.8	7	1258	0.35	7.3
8	*650*	*0.2*	*6.6*	8	1418	0.42	6.9
9	577	0.29	4.0	9	1690	0.42	8.2
10	523	0.19	5.6	10	1250	0.33	7.7
11	575	0.26	4.5	11	1232	0.32	7.8
12	550	0.25	4.5	12	1246	0.32	7.9
13	437	0.18	4.9	13	1162	0.31	7.6
14	*790*	*0.22*	*7.3*	14	1250	0.38	6.7
15	533	0.41	2.6	15	1088	0.34	6.5
16	685	0.41	3.4	16	1002	0.26	7.8
17	374	0.15	5.1	17	932	0.31	6.1
18	682	0.36	3.8	18	1396	0.37	7.7
19	879	0.4	4.5	19	1354	0.41	6.7
20	755	0.43	3.6	20	1924	0.44	8.9
21	901	0.38	4.8	21	1780	0.48	7.5
22	755	0.38	4.0	22	1274	0.39	6.6
23	919	0.38	4.9	23	980	0.28	7.1
24	1008	0.4	5.1	24	1130	0.3	7.7
25	*748*	*0.25*	*6.1*	25	1170	0.35	6.8
26	547	0.35	3.2	26	1224	0.38	6.5
27	687	0.45	3.1	27	1120	0.46	6.2
28	721	0.54	2.7	28	1114	0.27	8.4
29	750	0.39	3.9	29	1148	0.31	7.5
30	724	0.44	3.3	30	1454	0.4	7.4
31	527	0.31	3.5	31	1248	0.4	6.3
32	599	0.3	4.1	32	1255	0.36	7.1
33	747	0.49	3.1	33	1150	0.37	6.3
34	535	0.28	3.9	34	748	0.49	6.5
35	868	0.38	4.6	35	844	0.43	5.9

36	651	0.29	4.6	36	914	0.25	7.4
37	786	0.43	3.7	37	1294	0.39	6.7
38	689	0.31	4.5	38	1320	0.37	7.2
39	833	0.37	4.6	39	1186	0.33	7.3
40	666	0.33	4.1	40	1382	0.39	7.2
41	760	0.37	4.2	41	1150	0.35	6.7
42	706	0.26	5.5	42	2044	0.45	8.1
43	781	0.33	4.8	43	1878	0.63	6.1
44	1052	0.36	5.9	44	1216	0.4	6.2
45	814	0.21	7.9	45	1216	0.37	6.7
46	558	0.18	6.3	46	1150	0.37	6.3
47	793	0.37	4.4	47	1390	0.45	6.3
48	730	0.4	3.7	48	1118	0.33	6.9
49	1265	0.52	4.9	49	914	0.47	5.2
50	600	0.34	3.6	50	1300	0.35	7.5
M ± m	702.4±24	0.334±0,013	4.45±0.16		1259.9±38.1	0.376±0,01	6.81±0.16

The tables S7 and S8 are adopted from our paper: Ilyechova EY et al. A low blood copper concentration is a co-morbidity burden factor in Parkinson's disease development [131].
